# Serological Studies of Neurologic Helminthic Infections in Rural Areas of Southwest Cameroon: Toxocariasis, Cysticercosis and Paragonimiasis

**DOI:** 10.1371/journal.pntd.0000732

**Published:** 2010-07-06

**Authors:** Agathe Nkouawa, Yasuhito Sako, Sonoyo Itoh, Alida Kouojip-Mabou, Christ Nadège Nganou, Yasuaki Saijo, Jenny Knapp, Hiroshi Yamasaki, Minoru Nakao, Kazuhiro Nakaya, Roger Moyou-Somo, Akira Ito

**Affiliations:** 1 Department of Parasitology, Asahikawa Medical College, Asahikawa, Hokkaido, Japan; 2 Medical Research Center, Institute of Medical Research and Medicinal Plants Studies (IMPM), Ministry of Scientific Research and Innovation, Yaoundé, Cameroon; 3 Cité des Palmiers Health District, Douala, Cameroon; 4 Department of Parasitology and Infectious Diseases, Faculty of Medicine and Biomedical Sciences, University of Yaounde I, Yaoundé, Cameroon; 5 Division of Community Medicine and Epidemiology, Department of Health Science, Asahikawa Medical College, Asahikawa, Hokkaido, Japan; 6 Department of Parasitology, National Institute of Infectious Diseases, Tokyo, Japan; 7 Animal Laboratory for Medical Research, Asahikawa Medical College, Asahikawa, Hokkaido, Japan; University of California San Diego School of Medicine, United States of America

## Abstract

**Background:**

Both epilepsy and paragonimiasis had been known to be endemic in Southwest Cameroon. A total of 188 people (168 and 20 with and without symptoms confirmed by clinicians, respectively, 84.6% under 20 years old) were selected on a voluntary basis. Among 14 people (8.3%) with history of epilepsy, only one suffered from paragonimiasis. Therefore, we challenged to check antibody responses to highly specific diagnostic recombinant antigens for two other helminthic diseases, cysticercosis and toxocariasis, expected to be involved in neurological diseases. Soil-transmitted helminthic infections were also examined.

**Methodology/Principal Findings:**

Fecal samples were collected exclusively from the 168 people. Eggs of *Ascaris lumbricoides*, *Trichuris trichiura* and hookworms were found from 56 (33.3%), 72 (42.8%), and 19 (11.3%) persons, respectively. Serology revealed that 61 (36.3%), 25 (14.9%) and 2 (1.2%) of 168 persons showed specific antibody responses to toxocariasis, paragonimiasis and cysticercosis, respectively. By contrast, 20 people without any symptoms as well as additional 20 people from Japan showed no antibody responses. Among the 14 persons with epilepsy, 5 persons were seropositive to the antigen specific to *Toxocara*, and one of them was simultaneously positive to the antigens of *Paragonimus*. The fact that 2 children with no history of epilepsy were serologically confirmed to have cysticercosis strongly suggests that serological survey for cysticercosis in children is expected to be useful for early detection of asymptomatic cysticercosis in endemic areas.

**Conclusions/Significance:**

Among persons surveyed, toxocariasis was more common than paragonimiasis, but cysticercosis was very rare. However, the fact that 2 children were serologically confirmed to have cysticercosis was very important, since it strongly suggests that serology for cysticercosis is useful and feasible for detection of asymptomatic cysticercotic children in endemic areas for the early treatment.

## Author Summary

A total of 188 people (168 and 20 with and without symptoms confirmed by clinicians, respectively, 84.6% under 20 years old) were selected on a voluntary basis in Cameroon. Soil transmitted helminthic infections were prevalent among persons surveyed as is common in developing countries, since eggs of *Ascaris lumbricoides*, *Trichuris trichiura* and hookworms were found from 56 (33.3%), 72 (42.8%) and 19 (11.3%) persons, respectively. Serological analyses revealed that 61 (36.3%), 25 (14.9%) and 2 (1.2%) persons were positive to the diagnostic antigens specific for toxocariasis, paragonimiasis and cysticercosis, respectively. Among 14 people with epilepsy, 5 persons were seropositive to the antigen of *Toxocara* and one of them was simultaneously positive to the antigens of *Paragonimus*. Serological confirmation of cysticercosis in two children is very important, and we suggest that further serologic surveys of cysticercosis be carried out in both children and adults in this area for the promotion of a better quality of life including control and early treatment.

## Introduction

Parasitic infections are serious public health problems in many developing countries [1],[2]. These diseases can affect various tissues and organs including the brain leading to neurological dysfunction. Cysticercosis caused by *Taenia solium* metacestodes has been assumed to be the most common parasitic infection of the brain worldwide including Cameroon [3]–[5]. As cysticercosis is one of the major causative agents of the late-onset of epilepsy, the major work on cysticercosis has been carried out for adults but not for children in endemic areas, and other causative agents of epilepsy still remain unclear. Therefore, we were lead to obtain more information on the causative agents of epilepsy in developing countries, since many helminthic diseases including toxocariasis, paragonimiasis, onchocerciasis etc., and also protozoan diseases including malaria, toxoplasmosis and others may cause epilepsy [4]–[6]. Among these neglected helminthic diseases, toxocariasis is expected to have cosmopolitan distribution, since dogs and cats are companion animals with close contact with people in the world [7], [8]. Although there are no data on the prevalence of human toxocariasis in Cameroon, its prevalence in dogs in Cameroon is high [9]. Simultaneously, there is poor information on cysticercosis in children in Cameroon, although it seems to be rather common in the adult population [4], [5].

**Figure 1:**
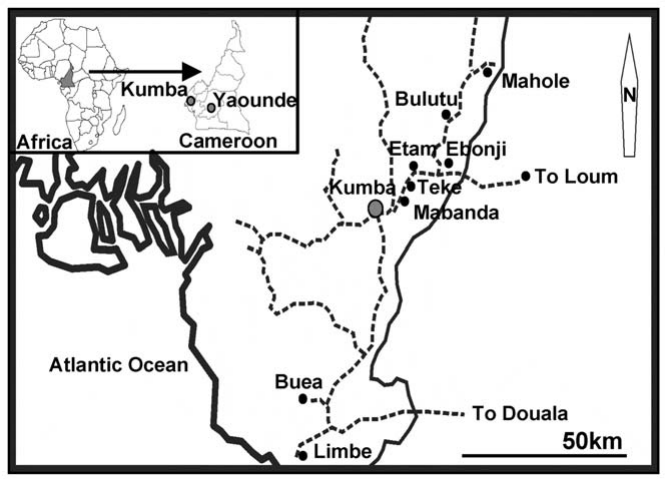
Locations of Bulutu, Ebonji, Etam and Teke in Tombel sub-Division, Southwest Province, Cameroon.

Tombel health district in South West Province in Cameroon (Figure 1) is known as an endemic focus of epilepsy and is also highly endemic for paragonimiasis [10],[11]. Our previous report in this area showed that 8.3% of enrolled people (14/168) suffered from epilepsy but only one of the epileptic patients simultaneously suffered from paragonimiasis [11]. Therefore, we concluded that paragonimiasis was not the major cause of epilepsy in children in this area.

In this study, we used the same 188 samples examined for paragonimiasis [11] and additional 20 samples from Japan, where cysticercosis and paragonimiasis have long been eradicated and toxocariasis is very rare [12], as healthy controls. We performed serosurveys using highly specific recombinant antigens for toxocariasis and cysticercosis, and simultaneously analyzed the unpublished data on microscopic observation of soil-transmitted helminthic (STH) infections. Serological data on paragonimiasis for this study were modified from published data [11]. Although onchocerciasis was known to be endemic in Cameroon and might be involved in neurological disorder, we could not examine simply because the lack of serological tools [13], [14].

## Materials and Methods

### Study sites

Four villages in rural areas, Bulutu, Ebonji, Etam and Teke, were selected for this study. They are located in the Tombel Health District (50,000–100,000 inhabitants) in the rain forest zone about 40 km northwest of Kumba, Manengouba Department, South West Province of Cameroon (4°3′N, 9°3′W). The annual average temperature is 24°C and the relative humidity varies from 52% to 74%. Agriculture is the principal economic activity; hunting and fishing are also practiced (Figure 1).

### Ethical statement

The survey, approved by the National Ethics Committee of Cameroon, was conducted in the general population in January 2004 and February 2006 in villages mentioned above.

### Human samples

The chief of each village was informed about the study and participants or parents/guardians were asked to give informed consent for participation. A total of 188 people ranged in age from 4 to 78 years (14.9±7.8 years in males and 13.1±6.1 years in females) were examined by clinicians and were asked whether they had experienced symptoms such as cough, haemoptysis, headache, epilepsy, chest pain, and eye disorder and whether they consumed raw and/or undercooked fresh water crabs or pork. Our study population with symptoms ranged from 0–10 years (80 persons), 11–20 years (63 persons), and >21 years (25 persons). Following the questionnaire, serum, sputum and fecal samples were collected from 168 people who accepted to participate to the study voluntarily (28, 52, 55 and 33 from Bulutu, Ebonji, Etam, and Teke villages, respectively). By contrast, 20 healthy persons [5 persons from each village including 11 females and 9 males ranged from 6 to 34 years (13.0±3.7 in males and 15.1±7.5 in females)] confirmed by clinicians donated serum samples exclusively; these serum samples were used as expected healthy controls. An additional 20 serum samples from students at Asahikawa Medical College (AMC), Japan, were used as confirmed healthy controls. Sputum was examined for eggs of *P. africanus* [11]. Fecal samples were examined by flotation techniques for the presence of eggs to provide a diagnosis of helminthic infections.

### Serology

A total of 208 serum samples were examined by ELISA. A recombinant antigen of *T. canis* second-stage larvae (0.5 µg/ml) was used for toxocaraisis [15]. Glycoproteins (GPs) (1.0 µg/ml) from *T. solium* cyst fluid purified by preparative isoelectric focusing (pH 9.2–9.6) were used for screening of cysticercosis by ELISA [16]. Immunoblot using a recombinant chimeric antigen, 100% specific to cysticercosis (0.5 µg/mini gel) was applied for serological confirmation of cysticercosis [16]–[19]. Somatic antigens of *P. africanus* adult worms (5µg/ml), which showed few cross reactivity with other parasitic infections were used for paragonimiasis [11]. Briefly, 96-well microtiter plates (Maxisorp; Nunc, Roskilde, Denmark) were coated with each of the antigens described above in PBS and incubated at 4°C overnight. The plates were probed with diluted serum samples. Serum dilutions were in 1∶200 with bicarbonate buffer for toxocariasis, and 1∶100 and 1∶200 with blocking buffer for cysticercosis and paragonimiasis, respectively, according to the original papers for these diseases described above. Peroxidase-conjugated rec-Protein G (Zymed, San Francisco, USA) diluted in 1∶1000 with blocking buffer was added into each well. Peroxidase activity was revealed by adding 0.4 mmol/l 2,2-azino-bis 3 ethybenz-thiazoline-6-sulphonic acid in 0.1 mol/l sodium citrate buffer, pH 4.7 containing 0.003% H_2_O_2_ at room temperature. The optical density (OD) was monitored at 405 nm on a microplate reader (ImmunoMini, model NJ-2300; Nalgene Nunc International, Tokyo, Japan). The cut-off value was calculated for each antigen based on the means+3SD of 40 healthy donors from the local areas in Cameroon (n = 20) and from Japan (n = 20).

### Statistical analyses

To obtain adjusted odds ratios (ORs) of paragonimiasis and toxocariasis seropositivities for each symptom, we performed multivariate logistic regression analysis adjusted for age (−10, 11–20, 21year) and sex. Because the number of cysticercosis seropositivity was rather small (n = 3), we did not analyze the ORs of cysticercosis seropositivity. For all statistical analyses, a 5% level of significance was applied. All statistical analyses were conducted using SPSS for Windows version 18.0 (SPSS, Inc., Chicago, U.S.A.).

## Results and Discussion

In this study, the samples used for paragonimiasis [11] were also tested for toxocariasis and cysticercosis and also the data of STHs were analyzed. The enrolled persons (168: 78 males and 90 females) were diagnosed suffering from cough (n = 135, 80.3%), haemoptysis (n = 18, 11.3%), chest pain (n = 80, 47.6%), epilepsy (n = 14, 8.3%), visual impairment (n = 30, 17.8%) and headache (n = 106, 63.0%) and had histories of eating raw or undercooked crabs (n = 137, 81.5%) or pork (n = 135, 80.3%). Microscopic examination revealed *Paragonimus* eggs in sputum from 16 (9.5%) persons but no eggs from feces [11], whereas *A. lumbricoides*, *T. trichiura*, and hookworms were found in feces from 56 [33.3%; 30 (53.5%) males and 26 (46.4%) females], 72 [42.8%; 38 (52.7%) males and 34 (47.2%) in females] and 19 [11.3%; 14 (73.6%) males and 5 (26.3%) females] persons, respectively. Among these helminthic infections, hookworm infection exclusively showed statistically significant difference between the genders (p<0.05). The difference in prevalence between males and females for hookworm infection may be due to the barefoot roaming behavior of males but further investigation of this topic is needed. The highest multiple infections were found in 3 kids infected with 3 STHs and were simultaneously seropositive for paragonimiasis and toxocariasis as well.

**Figure 2:**
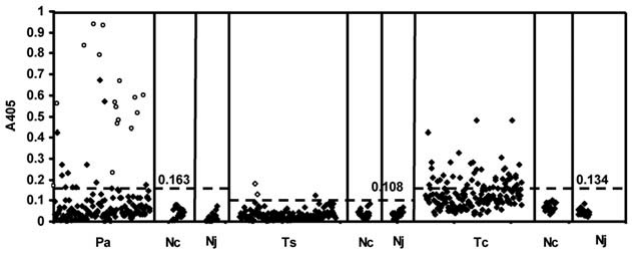
ELISA results for paragonimiasis (Pa), cysticercosis (Ts) and toxocariasis (Tc) from 208 persons. The surveyed persons (n = 208) include 168 and 40 (20 persons from Cameroon (Nc) and 20 from Japan (Nj)) with and without symptoms, respectively. **○**, samples with *Paragonimus* eggs in the sputum. Serology could detect more cases than microscopical examination of sputum and was expected to be more sensitive for detection of paragonimiasis including immature adult stage [11]. **◊**, samples positive against the recombinant antigen 100% specific to cysticercosis by immunoblot [17]. The broken line denotes the respective cut-off value for each disease and each cut-off value is shown.

ELISA for diagnosis of toxocariasis, paragonimiasis and cysticercosis indicated that 61 [36.3%; 31 (50.8%) males and 30 (49.1%) females], 25 [14.9%; 10 (40%) males and 15 (60%) females] [11], and 3 [1.8%; 2 boys of 13 and 11 year-old, and one girl, 4 year-old] persons were positive (Figure 2). Persons with cough and haemoptysis were more likely to have paragonimiasis (Table 1, OR = 7.19 and 2.28 respectively, p<0.001), whereas there was a relative risk with other symptoms. As none of the symptoms were specific for toxocariasis, the probability to have the infection was equally likely in exposed and control group as OR values were close to 1 (Table 1). The likelihood for cysticercosis to occur was not included due to the low number of seropositive persons. Nonetheless, there were crucial differences in antibody responses between the two groups. Furthermore, there was no difference in OD values between healthy controls from endemic Cameroon and from non-endemic, Japan where we expected no positive samples from students at AMC. Therefore, we concluded that the serological findings indicated specific responses to these three helminthic infections. The ELISA system applied for paragonimiasis in this study was much more sensitive for diagnosis than detection of eggs as already shown (Figure 2) [11]. As it has already been shown that the ELISA for toxocariasis in this study showed no cross-reactions with ascariasis patients in Asia and Latin America [20], [21], we consider that it is highly specific to toxocariasis. As children are the most risky population for toxocariasis and the prevalence of *T. canis* in dogs in Cameroon was very high [9], we expected that 61 persons (36.3%) were really exposed to eggs of *Toxocara* [22], [23]. Among these seropositive persons, 11 persons were concluded to have dual infection of both *Toxocara* and *Paragonimus*.

**Table 1:** Odds ratio of positive serological test for each symptom among 188 subjects.

Type of symptom	Serological test	Odds ratio*	95%CI	P value
Headache	Paragonimiasis	1.22	0.52–2.87	0.999
	Toxocariasis	1.12	0.60–2.08	0.729
Haemoptysis	Paragonimiasis	7.19	2.68–19.30	<0.001
	Toxocariasis	0.99	0.40–2.46	0.977
Cough	Paragonimiasis	2.28	0.50–10.29	0.284
	Toxocariasis	1.97	0.75–5.16	0.167
Chest pain	Paragonimiasis	1.44	0.61–3.43	0.406
	Toxocariasis	1.33	0.71–2.48	0.371
Eye disorder	Paragonimiasis	0.91	0.23–3.64	0.910
	Toxocariasis	1.38	0.06–4.31	0.521
Epilepsy	Paragonimiasis	0.51	0.52–3.70	0.539
	Toxocariasis	1.30	0.39–4.28	0.667

*Adjusted for age (−10y, 11–20y, >21y) and sex.

**Figure 3:**
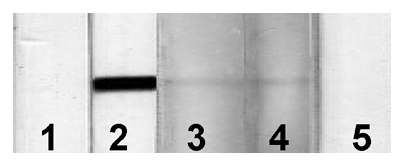
Immunoblot using the recombinant antigen of the 3 samples showing weak positive response by ELISA. Sera were in 1∶20 dilutions. Lane 1: negative control, lane 2: positive control, lanes 3–5: samples exhibited weak positive response by ELISA. Lanes 3 and 4 corresponding to the samples showing higher OD value by ELISA were positive to the recombinant antigen by immunoblot.

Three children (1.7%) showing weak responses to the GPs of *T. solium* by ELISA (Figure 2) were further analyzed using the recombinant antigen for serological confirmation of cysticercosis, since there are no false positive antibody responses to the recombinant antigen by immunoblot [17]–[19]. Two of them showing higher OD values by ELISA (Figure 2) exhibited positive response with the recombinant antigen by immunoblot (Figure 3) [17], [18]. Therefore, these two cases are considered as asymptomatic cysticercosis and are important targets for cysticercosis studies in the future. We believe that further epidemiological surveys for neurocysticercosis in the adult population should be carried out in the same areas, since 1) the late-onset epilepsy due to cysticercosis is expected to be detectable more common from senior people [3]–[6], [24], [25], 2) cysticercosis prevalence in Cameroon ranges from 2.5% to 13% [4], [5] and 3) more than the half of epileptic adult patients show antibodies against cysticercosis in West and North West regions in Cameroon using the same serology [5].

In Papua, Indonesia, one of the most serious endemic areas of cysticercosis in the world, more than 80% and 70% of people over 18 years old, who had history of epileptic seizures with or without subcutaneous nodules, were confirmed as having cysticerci, respectively [26], [27]. Approximately 30% of asymptomatic healthy people were serologically identified as positive for cysticercosis and follow up investigations revealed that many of them had detectable subcutaneous nodules. Furthermore, the most recent retrospective study using molecular tools has revealed that a cysticercus of *T. solium* survived at least for 10 years in a patient's brain [28].

According to these data mentioned above, the most important implication on cysticercosis from this serological study is that asymptomatic cysticercosis can be detected from children in endemic areas. Therefore, introduction of serological screening of children becomes highly informative for detection of asymptomatic cases and for getting better and early treatment for them [29]. Follow-up studies on these 2 boys using neuroimaging tools are necessary for further evaluation. We recommend highly reliable serological screening for cysticercosis for all pupils in the primary school, if possible, or all teenagers at least in highly endemic areas. As risk factors associated with human cysticercosis include the occurrence of cysticercosis in pigs, detection of adult worm carriers should be investigated. For the future survey of taeniasis carriers, both copro-ELISA [30] and copro-DNA tests [31] are expected to be introduced in this area, Cameroon, and in any other areas where cysticercosis is highly endemic.

Participants in the study were selected on a voluntary basis and may not be representative for the population as the whole but the numbers of children younger than 20 years were approximately 84.6% of surveyed persons. Therefore, the results are highly informative as a preliminary study identifying areas for further investigation of all these helminthic infections in this area.

In conclusion, toxocariasis, paragonimiasis and cysticercosis have been serologically confirmed among surveyed persons. Five of 14 epilepsy cases were sero-positive for toxocariasis. Correlation between epilepsy and these helminthic infections should be further evaluated, since screening of children for these parasitic diseases may become more important and feasible for the early treatment and prevention of these infections and promotion of better quality of life in the future.
